# Multicomponent body composition of university club sport athletes

**DOI:** 10.1080/15502783.2024.2446575

**Published:** 2024-12-26

**Authors:** Dale R. Wagner, Edward M. Heath, Sara A. Harper, Elizabeth A. Cafferty, Masaru Teramoto, Alyssa Evans, Tate Burch, Jacob McBride, Steven Spencer, Michael N. Vakula

**Affiliations:** a Utah State University, Kinesiology & Health Science, Logan, UT, USA; b University of Alabama in Huntsville, Kinesiology, Huntsville, AL, USA; c University of Utah, Physical Medicine & Rehabilitation, Salt Lake City, UT, USA; d Utah State University, Nutrition, Dietetics & Food Sciences, Logan, UT, USA

**Keywords:** Body fat, fat-free mass index, multicompartment, norms, sports

## Abstract

**Background:**

The body composition of National Collegiate Athletic Association (NCAA) athletes is well documented but no such data exist for university club sports athletes. Additionally, the majority of norms for NCAA athletes were created from individual methods requiring assumptions.

**Objective:**

This study used a four-component (4C) model to measure the body composition of university club sports athletes.

**Methods:**

Data were collected on club athletes participating in baseball, climbing, cycling, figure skating, gymnastics, ice hockey, lacrosse, pickleball, powerlifting, racquetball, rodeo, rugby, soccer, swimming, ultimate, and volleyball. The 4C model consisted of body volume, total body water, and bone mineral content measured by air displacement plethysmography, bioimpedance spectroscopy, and dual-energy x-ray absorptiometry, respectively. Percentile ranks were created for body fat percentage (%BF) and fat-free mass index (FFMI). Mean differences across teams were quantified with Cohen’s *d*.

**Results:**

In total, 225 athletes (137 men, 88 women) completed data collection. Athletes varied in competitive experience (1 to 22 y) and body mass index (16.9 to 36.4 kg·m^−2^). The density of the FFM was significantly greater than the assumed value of 1.100 g·cm^−3^ for both men (*p* = .043) and women (*p* = .011). The %BF ranged from 4.9% to 35.7% (14.3 ± 5.8% BF) for men and from 15.5% to 42.8% (25.2 ± 6.0% BF) for women. FFMI ranged from 15.6 kg·m^−2^ to 26.8 kg·m^−2^ (30.0 kg·m^−2^ outlier removed) for men and from 14.1 kg·m^−2^ to 22.6 kg·m^−2^ for women. Differences across sports in %BF and FFMI were considered large-sized effects (*d* ≥ 0.80) for both men and women. Weight-sensitive sports (e.g. cycling and climbing) had the lightest athletes and were among the leanest, whereas power athletes (e.g. powerlifting and rugby) were among the heaviest athletes and had the highest FFMI.

**Conclusions:**

Differences in %BF and FFMI are evident across sports. Due to the small sample size, use caution when interpreting the data as reference values for club sports athletes.

## 1. Introduction

Body composition, including the proportion of body fat (%BF) relative to total mass, as well as the quality of bone and the amount of lean tissue one has, is an important component of health [1] and improves the predictive accuracy of resting energy expenditure among athletes [2]. Many variables such as skill and psychology influence athletic performance. Body composition is an additional variable that is influential to athletic success in many sports, including gravitational sports in which mass restricts movement, sports with weight classes, and aesthetic sports with higher performance scores often corresponding to a perceived ideal body shape [3,4]. A higher fat mass (FM) is related to slower race times, whereas a higher fat-free mass (FFM) is advantageous for strength and power performance [4]. Increasingly, the FFM index (FFMI), a height-adjusted metric of FFM, is used to assess an athlete’s capacity for lean mass accretion and as a marker of athletic potential [5–8]. Given the importance of body composition to both health and athletic performance, it is not surprising that norms or reference values for body composition have been published for the general population [9–12] as well as National Collegiate Athletic Association (NCAA) athletes [5,6,8,13–15].

In the United States, numerous university students participate in athletic competitions unaffiliated with the NCAA in the form of “club sports.” Unlike NCAA athletes, club sports athletes receive no scholarships, and the teams receive only minimal financial support from the university. Although club sports are largely financially self-sustaining, the athletes compete against club sports athletes from other universities, train regularly, and typically receive coaching. Most club sports are well organized with conferences and regional and national championships. In informal conversations with club sports athletes, we found that there are numerous reasons why university students choose to participate in sport at the club level rather than as an NCAA athlete. Many students participate in a club sport rather than an NCAA sport because the university they are attending does not offer their sport of interest at the NCAA level. Other club sports athletes were either not good enough to make an NCAA team or do not want to commit the time required to be on an NCAA team, yet they still want to compete at a higher level than intramural sport. McKay et al. [16] developed a classification framework to characterize training and performance status ranging from 0 (sedentary) to 5 (world class). They placed NCAA Division I athletes at tier 4. Using this classification metric, club sports athletes would best fit at either tier 3 (skill level equivalent to NCAA Division III) or tier 2 (training ~3 times per week with a purpose to compete in a specific sport). Table 1 summarizes the similarities and contrasts of club sports with NCAA sports. Club sports range from traditional team sports (e.g. soccer and volleyball) to olympic sports (e.g. cycling and powerlifting) to less traditional sports (e.g. ultimate and pickleball). It is estimated that there are about 10 times more club sport athletes than NCAA Division I athletes [17,18]; however, to date, no body composition data exists for university club sports athletes.

**Table 1.** Club sports compared to different levels of NCAA sports.

Variable	University Club Sport	NCAA DIII Sport	NCAA DII Sport	NCAA DI Sport
Age of athletes	Late teens to early twenties	Late teens to early twenties	Late teens to early twenties	Late teens to early twenties
Years of competitive experience	Varies widely from sport to sport	Typically >10 years	Typically >10 years	Typically >10 years
Team selection	Typically involves competitive tryouts among interested students already enrolled at the university	Typically recruited based on strong high school play; relaxed recruitment rules relative to DI and DII programs; university sets eligibility requirements	Typically recruited based on excellent high school play; strict recruitment and eligibility requirements set by NCAA	Typically recruited based on exceptional high school play; strict recruitment and eligibility requirements set by NCAA
Level of competition	More skilled and competitive than intramural sport; competitive with NCAA DIII programs	High level of skill and competition, but less than NCAA DII	High level of skill and competition, but less than NCAA DI	Highest level of sport with the exception of professional and international athletes
Structured team training and/or conditioning	Typically 2–3 days/week (5–10 hours/week)	Daily, but balanced with academics (20 hours/week)	Daily; high time commitment (25 hours/week)	Daily; very high time commitment (30 hours/week: practice, meetings, competition, conditioning, extensive travel)
Coaching	Part-time; pro bono or minimal compensation	Full-time, but often with other responsibilities (e.g. teaching)	Full-time coach; similar to academic salaries	Full-time coach; highly paid
Athlete payment	None	No athletic scholarships; academic or need-based scholarships are common	Athletic scholarships (often partial tuition)	Athletic scholarships (often full tuition); NIL (name, image, and likeness) money is more prevalent
University size	Varies; all sizes	Usually small; median undergraduate enrollment of 1,751	Mostly small to mid-size; median undergraduate enrollment of 2,428	Typically large; median undergraduate enrollment of 8,960
University financial support	Student-funded	University-funded; very small athletic budget	University-funded; small athletic budget relative to DI programs	University-funded; large athletic budget
Facilities for training and competition	Reserved shared-space with student/campus recreation	Exclusive use for NCAA athletes; some shared space with academics	Exclusive use for NCAA athletes	Exclusive use for NCAA athletes; large seating capacity
Governing body	Each sport has its own national governing body	NCAA	NCAA	NCAA
Participation	Approx. 2,000,000 [17]	Approx. 200,000 [18]	Approx. 122,000 [18]	Approx. 190,000 [18]

Typically, normative values for body composition are created from laboratory methods of body composition assessment such as dual-energy x-ray absorptiometry (DXA) [5,6,10–14] or air displacement plethysmography (ADP) [8,15] or field methods such as bioimpedance analysis (BIA) [9,19] or skinfolds [20,21]. However, these individual body composition methods require certain assumptions about the FFM [22]. For example, a common body composition model is the two-component model, in which body mass (BM) = FM + FFM, with assumptions made about the proportions of the FFM (e.g. water content and bone content). In contrast, the multicomponent model of body composition combines multiple body composition methods to eliminate or minimize assumptions. For example, a four-component (4C) model, as follows: BM = FM + water + bone + residual, accounts for individual variability in the water and bone proportions of the FFM. Because more is measured and less is assumed, 4C models are considered more accurate than individual body composition methods [22–24]. Indeed, the 4C body composition model is recommended for athletes [24,25]. While the 4C model has been utilized for small samples of athletes, there are no known large 4C body composition athlete samples. Thus, the purpose of this study was to use a 4C body composition model to evaluate the body composition of university club sports athletes.

## 2. Materials and methods

### 2.1. Participants

Participants were athletes, ≥18 y of age competing on a university club sports team. Men were recruited from 16 club sports (baseball, climbing, cycling, gymnastics, ice hockey, jump rope, lacrosse, pickleball, powerlifting, racquetball, rodeo, rugby, soccer, swimming, ultimate, and volleyball). Women were recruited from 13 club sports (climbing, cycling, figure skating, gymnastics, jump rope, lacrosse, powerlifting, rodeo, rugby, soccer, swimming, ultimate, and volleyball). In an effort to maximize participation and minimize recruitment bias within teams, money was added to the teams’ budgets at the rate of $10 per participant up to a maximum of $200 per team. Athletes were measured near the start of their competitive season. Self-reported exclusion criteria included current pregnancy, a pacemaker or other implanted cardiac device, surgical metal implant, and loss of limb(s). Additionally, athletes with a height of >194 cm were excluded due to the size limitation of the DXA table. The study was approved by Utah State University’s Institutional Review Board (protocol #12253), and all participants gave written informed consent prior to their participation.

### 2.2. Procedures

All measurements on an individual took place in a single session. Participants were given the following pretesting instructions in an attempt to ensure euhydration: no vigorous exercise for 12 h prior, no major meals for 4 h prior, and drink 20 oz (591 mL) of water approximately 2 h prior to appointment. Upon arrival at the lab, participants emptied their bladder and provided a mid-stream urine sample. Urine specific gravity (USG) was measured with a digital refractometer (Palm Abbe, Misco, Cleveland, OH).

Height was measured with a wall-mounted stadiometer (Seca 216, Seca Corp., Ontario, CA) to the nearest 0.1 cm. Body mass (BM) was measured to the nearest 0.01 kg with the Bod Pod scale (Cosmed USA, Inc., Concord, CA) as part of the ADP testing procedure. Men wore compression shorts, and women wore compression shorts and a sports bra for all procedures.

The Bod Pod was calibrated with the manufacturer-provided calibration cylinder. Body volume (BV) and body density (D_b_) were measured using the Bod Pod ADP system according to the manufacturer’s instructions. This included sitting in the Bod Pod for at least two trials of 20 s each to achieve BV measurements that agree to within 150 mL. Thoracic gas volume (TGV) was measured rather than predicted to maximize the accuracy of the method [26]. If the participant could not achieve a valid TGV test after 5 trials, the predicted TGV was used.

The DXA was calibrated with the manufacturer-provided phantom calibration block. Bone mineral content (BMC) was measured with a whole-body DXA scan (Horizon-W, Hologic, Inc., Marlborough, MA; APEX system software version 5.6.0.5). Participants rested in a supine position on the scanner table, consistent with the manufacturer’s procedures for the 6-min total body anteroposterior scan.

The bioimpedance spectroscopy (BIS) machine was calibrated with the manufacturer-provided test cell. BIS (SFB7, ImpediMed, Inc., Carlsbad, CA) was used to measure total body water (TBW). Participants were supine on a nonconducting treatment table for 5 min before testing. Arms and legs were extended and slightly abducted so as not to be in contact with other body parts during the test. The skin was cleaned with an alcohol swab, and electrodes were placed in a tetrapolar configuration (right hand, wrist, ankle, and foot). The manufacturer’s dual-tab electrodes were used to ensure a 5 cm distance between distal and proximal electrodes.

### 2.3. Statistical analyses

Prior to data analysis, %BF was calculated using the formula of Wang et al. [27]:

$$\mathrm{Fat}\left(\mathrm{kg}\right)=\left(2.748\;x\;\mathrm{BV}\right)-\left(0.699\;x\;\mathrm{TBW}\right)+\left[1.129\;x\;\left(\mathrm{BMC}\;x\;1.0436\right)\right]-\left(2.051\;x\;\mathrm{BM}\right)$$

This 4C molecular model was originally validated by summing major body elements (total body potassium, sodium, chlorine, and calcium) measured by in vivo neutron activation analysis. Wang et al. [27] reported a mean difference in soft tissue minerals between this molecular model and neutron activation analysis of just 20 g with a standard error of estimate (SEE) of 32 g.

Subsequent to the calculation of FM, other variables of interest were derived from simple mathematical calculations:

$$\%\mathrm{BF}=\left(\mathrm{FM}/\mathrm{BM}\right)\;x\;100$$

FFM=BM−FM

$$\mathrm{Fat}-\mathrm{free}\;\mathrm{mass}\;\mathrm{index}\left(\mathrm{FFMI}\right)=\mathrm{FFM}\left(\mathrm{kg}\right)/\mathrm{height}{\left(m\right)}^{2}$$

Additionally, the density of the FFM (D_FFM_) was calculated. First, following the procedures of Evans et al. [28], nonosseous mineral was assumed to be 23% of the DXA-measured BMC, and the DXA-measured BMC was multiplied by 1.0436 to get osseous mineral. Osseous and nonosseous minerals were summed to obtain the total body mineral. Next, the percentages of the FFM components (water, mineral, and protein or residual) were calculated by dividing the component by the FFM. Finally, the D_FFM_ was calculated from the fractions of water (%*w*), total body mineral (%*m*), and protein (%*p*), assuming the densities of the respective components [29], using the following equation:

$$D_{\mathrm{FFM}}=1/\left[\left(\%w/0.9937g\;\cdot \;{\mathrm{cm}}^{-3}\right)+\left(\%m/3.038g\;\cdot \;{\mathrm{cm}}^{-3}\right)+\left(\%p/1.34g\;\cdot \;{\mathrm{cm}}^{-3}\right)\right]$$

Descriptive statistics were calculated, and sex-specific norms (e.g. percentile ranks) for %BF and FFMI were created. Some investigators suggest that FFMI needs an additional height correction to account for greater body width and thickness of taller athletes [5–8]. Consequently, FFMI_raw_ values in all subjects above the median FFMI were regressed against height; FFMI_adj_ was calculated from the regression slope and mean height of all subjects (men = 179.3 cm; women = 166.6 cm) using the following equation [7]:

$$\mathrm{FFM}I_{\mathrm{adj}}=\mathrm{FFM}I_{\mathrm{raw}}+\left[\mathrm{slope}\;x\;\left(\mathrm{mean}\;\mathrm{ht}-\mathrm{subject}\;\mathrm{ht}\right)\right]$$

To estimate normative values of %BF and FFMI, nonparametric bootstrap confidence intervals (CIs) with 10,000 replications [30–32] of medians were computed for these two measures (excluding sports with *n* = 1 or 2), separately for sex and sport. Further, mean differences across teams for variables of interest were evaluated based on effect sizes, specifically using Cohen’s *d* [33]. The D_FFM_ and the proportions of the components that constitute the FFM were evaluated against Brozek’s [29] reference body assumptions of 1.100 g·cm^−3^ for D_FFM_, 73.8% water, 6.8% mineral, and 19.4% protein using a one sample t-test. All data were analyzed using SPSS version 25 (IBM, Inc., Armonk, NY) and Stata/MP 18.0 (StataCorp LLC, College Station, TX). Statistical significance was accepted at *p* < 0.05.

## 3. Results

In total, 225 athletes (137 men, 88 women) completed all aspects of data collection. Nearly all (89% men, 94% women) were Caucasian. Ninety percent of the sample had a USG of <1.025 (median = 1.011). Twelve percent of the sample was unable to perform the TGV maneuver even after 5 attempts; the predicted TGV was used for these individuals, and they remained in the data analysis. Descriptive and anthropometric data by sport and sex are in Table 2.

**Table 2.** Descriptive and anthropometric data (means ± SD) by sex and sport.

Sport (N)	Age (y)	Experience^a^ (y)	Ht (cm)	BM (kg)	BMI (kg·m^−2^)
**Men**					
Baseball (11)	21.2 ± 1.4	12.7 ± 2.3	178.8 ± 4.5	79.4 ± 5.8	24.8 ± 1.5
Climbing (10)	21.6 ± 2.0	2.0 ± 1.9	175.5 ± 7.3	68.9 ± 6.3	22.4 ± 1.3
Cycling (4)	21.8 ± 2.2	6.0 ± 3.4	180.9 ± 6.4	69.0 ± 6.8	21.1 ± 3.3
Gymnastics (6)	20.5 ± 2.2	6.0 ± 5.9	176.5 ± 5.5	70.8 ± 7.8	22.7 ± 1.7
Ice hockey (7)	21.3 ± 1.9	14.7 ± 4.8	178.5 ± 7.1	74.5 ± 9.4	23.4 ± 2.4
Lacrosse (26)	20.7 ± 1.8	7.1 ± 3.0	179.8 ± 7.4	84.0 ± 9.1	26.0 ± 2.1
Pickleball (3)	22.7 ± 1.5	2.3 ± 1.5	172.9 ± 7.2	76.1 ± 18.0	25.2 ± 4.3
Powerlifting (11)	21.0 ± 1.8	1.0 ± 0.2	176.3 ± 5.4	85.7 ± 12.4	27.6 ± 3.9
Racquetball (4)	22.0 ± 1.2	3.0 ± 3.4	183.2 ± 5.4	82.7 ± 22.2	24.5 ± 5.8
Rodeo (4)	24.0 ± 1.7	14.0 ± 5.6	175.5 ± 4.7	80.4 ± 12.0	26.2 ± 5.2
Rugby (10)	21.4 ± 1.9	2.8 ± 2.3	180.0 ± 5.0	92.0 ± 13.1	28.5 ± 4.6
Soccer (4)	22.3 ± 1.0	16.3 ± 2.1	177.7 ± 6.3	74.7 ± 11.1	23.6 ± 2.1
Swimming (5)	22.2 ± 3.0	11.0 ± 5.1	179.2 ± 1.7	84.1 ± 19.1	26.2 ± 6.1
Ultimate (13)	21.6 ± 1.3	5.1 ± 3.1	177.4 ± 7.2	73.6 ± 11.7	23.3 ± 2.8
Volleyball (19)	21.1 ± 1.9	5.4 ± 2.9	185.0 ± 5.4	79.7 ± 11.0	23.3 ± 3.0
Total (137) (range)	21.3 ± 1.8 (18–25)	6.6 ± 5.1 (1–22)	179.3 ± 6.7 (162.1–193.9)	79.6 ± 12.2 (57.3–116.0)	24.7 ± 3.5 (16.9–36.4)
**Women**					
Climbing (7)	20.6 ± 1.9	1.4 ± 1.1	161.6 ± 4.7	55.1 ± 6.0	21.1 ± 1.6
Figure skating (2)	20.0 ± 1.4	3.5 ± 2.1	168.5 ± 12.0	58.7 ± 13.7	20.5 ± 1.9
Gymnastics (5)	19.2 ± 2.2	6.0 ± 3.5	156.1 ± 6.9	57.6 ± 5.2	23.6 ± 1.6
Lacrosse (9)	18.7 ± 1.0	5.6 ± 2.7	165.8 ± 7.6	63.1 ± 9.4	22.8 ± 2.0
Powerlifting (4)	20.5 ± 2.5	2.5 ± 2.4	169.6 ± 8.0	81.8 ± 15.5	28.3 ± 3.7
Rodeo (5)	20.6 ± 1.9	11.2 ± 4.8	168.7 ± 3.2	66.6 ± 4.9	23.4 ± 2.0
Rugby (17)	20.9 ± 1.5	2.4 ± 2.5	167.1 ± 6.4	74.1 ± 13.1	26.5 ± 4.3
Soccer (7)	20.6 ± 1.5	14.4 ± 2.5	166.8 ± 5.3	64.6 ± 7.3	23.2 ± 2.3
Swimming (9)	19.2 ± 1.1	9.0 ± 2.4	168.5 ± 7.1	71.9 ± 11.9	25.3 ± 3.8
Ultimate (8)	20.8 ± 1.7	5.2 ± 2.4	165.8 ± 7.0	60.0 ± 10.7	21.8 ± 3.2
Volleyball (13)	20.2 ± 2.0	8.3 ± 2.2	171.0 ± 6.2	67.4 ± 8.3	23.0 ± 2.3
Total (88) (range)	20.2 ± 1.7 (18–24)	6.3 ± 4.5 (1–19)	166.6 ± 7.1 (149.2–180.5)	66.6 ± 11.7 (46.2–106.9)	23.9 ± 3.5 (18.1–34.8)

^a^Experience defined as years of competitive experience in the sport.

Note: A female cyclist and one male and one female jump roper also took part in the study. As the sole representatives for their sports, their data are not presented to protect their confidentiality.

**Figure 1.**
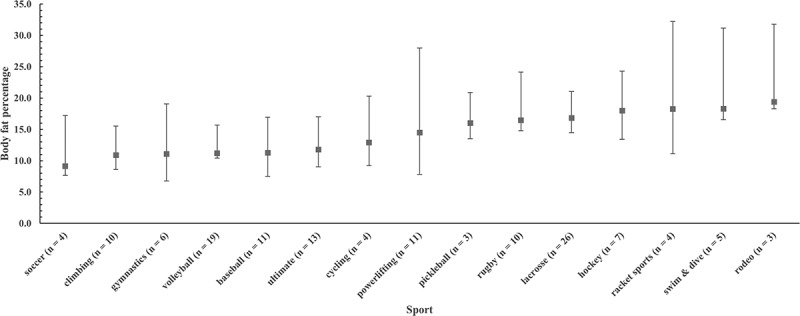
Plot of medians and 95% bootstrap confidence intervals for body fat percentage (%BF) for men by club sport. Sports are ranked by mean %BF from lowest (left) to highest (right).

**Figure 2.**
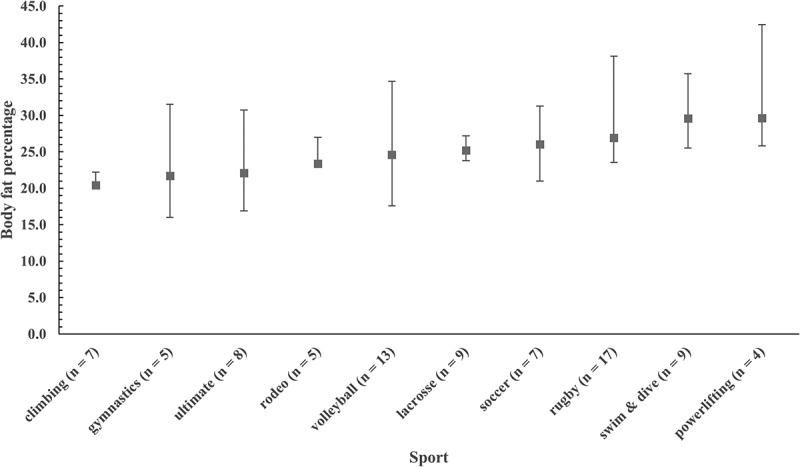
Plot of medians and 95% bootstrap confidence intervals for body fat percentage (%BF) for women by club sport. Sports are ranked by mean %BF from lowest (left) to highest (right).

For men, %BF ranged from 4.9% to 35.7% (mean ± SD: 14.3 ± 5.8% BF), and for women the values were 15.5% to 42.8% (mean ± SD: 25.2 ± 6.0% BF). Plots of medians and 95% bootstrap CIs for %BF by club sport are illustrated in Figures 1 and 2 for men and women, respectively. Traditional box plots with inter-quartile range are available as supplemental files. Percentile ranks for the %BF of men and women club sport athletes are in Table 3.

**Table 3.** Body fat percentage (%BF) percentiles in university club sports athletes.

Percentile	Men %BF	Women %BF
10	21.3	34.2
20	18.9	30.3
30	17.3	27.0
40	15.2	26.1
50	13.5	24.8
60	11.8	23.4
70	10.4	21.6
80	8.9	20.0
90	7.7	17.2

**Figure 3.**
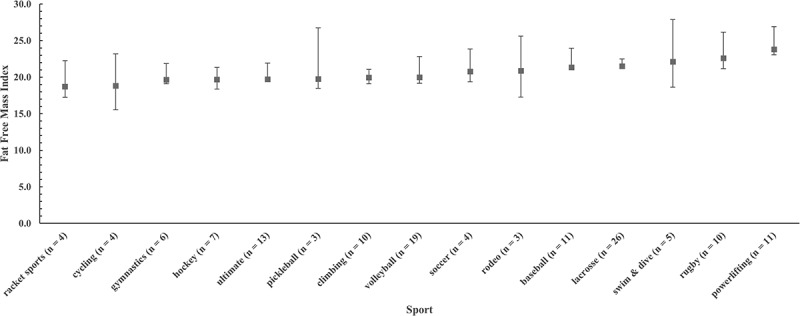
Plot of medians and 95% bootstrap confidence intervals for fat-free mass index (FFMI) for men by club sport. Sports are ranked by mean FFMI from lowest (left) to highest (right).

**Figure 4.**
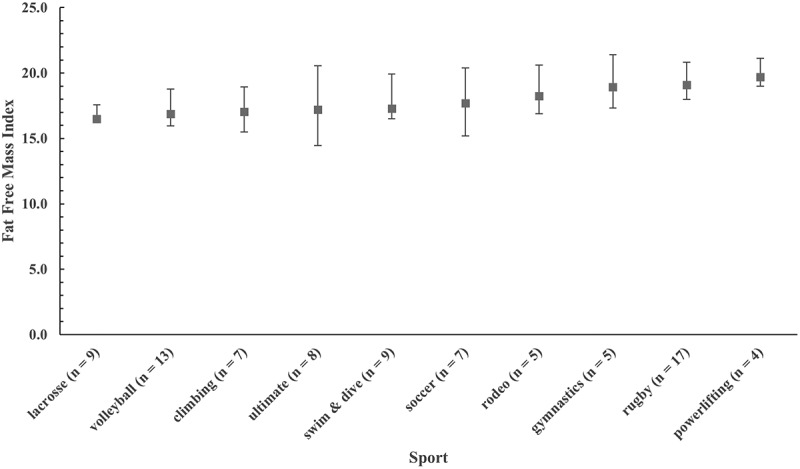
Plot of medians and 95% bootstrap confidence intervals for fat-free mass index (FFMI) for women by club sport. Sports are ranked by mean FFMI from lowest (left) to highest (right).

There was one statistical outlier (>3.2 SD) [34] with a FFMI of 30.0 kg·m^−2^, so this male powerlifter was removed from the FFMI analysis. Median FFMI for men was 21.0 kg·m^−2^. Regressing FFMI_raw_ against height for the subgroup of men with FFMI_raw_ > 21.0 kg·m^−2^ resulted in a nonsignificant (*p* = .551) slope of −0.020. Means for FFMI_raw_ and FFMI_adj_ were both 21.0 ± 2.2 kg·m^−2^ and not significantly different (*p* = .891). Median FFMI for women was 17.6 kg·m^−2^. Regressing FFMI_raw_ against height for the subgroup of women with FFMI_raw_ > 17.6 kg·m^−2^ resulted in a nonsignificant (*p* = .676) slope of −0.010. Means for FFMI_raw_ and FFMI_adj_ were both 17.8 ± 1.7 kg·m^−2^ and not significantly different (*p* = .987). Like Harty et al. [6], we found no significant difference between FFMI_raw_ and FFMI_adj_; thus, FFMI data and figures are reported as FFMI_raw_. For men, FFMI ranged from 15.6 kg·m^−2^ to 26.8 kg·m^−2^ (outlier removed), and the range for women was 14.1 kg·m^−2^ to 22.6 kg·m^−2^. Plots of medians and 95% bootstrap CIs for FFMI by sport are presented in Figures 3 and 4 for men and women, respectively. Traditional box plots with inter-quartile range are available as supplemental files. Table 4 provides percentile ranks for FFMI for both men and women club sports athletes.

**Table 4.** Fat-free mass index (FFMI) percentiles in university club sports athletes.

Percentile	Men FFMI (kg·m^−2^)	Women FFMI (kg·m^−2^)
10	18.4	15.7
20	19.1	16.4
30	19.7	16.9
40	20.2	17.3
50	21.0	17.6
60	21.4	18.2
70	22.0	18.6
80	22.7	19.2
90	23.8	19.8

Data by sex for BMC, BMD, %*w*, %*m*, %*p*, and D_FFM_ are in Table 5. BMD was moderately correlated with FFM in both men (*r* = .446) and women (*r* = .407). The %*w* was not significantly different between men and women (*p* = .281), but women had greater %*m* (*p* < .001) while men had greater %*p* (*p* = .010). The D_FFM_ was not significantly different between the sexes (*p* = .480). Both the men and women athletes in this study had significantly (*p* < .001) higher %*p* and lower %*w* and %*m* values than Brozek’s [29] assumed reference body values. Brozek’s [29] assumed D_FFM_ of 1.100 g·cm^−3^ was significantly less than the D_FFM_ for both the men (*p* = .043) and women (*p* = .011) in this sample.

**Table 5.** Multicomponent mode variables by sex. Data are means ± SD with minimum and maximum values in parentheses.

Variable	Men (*n* = 137)	Women (*n* = 88)
BMC (kg)	3.03 ± 0.40 (2.13–3.94)	2.33 ± 0.31 (1.53–3.10)
BMD (g·cm^−2^)	1.293 ± 0.102 (1.012–1.537)	1.174 ± 0.072 (0.991–1.354)
Water (% of FFM)	71.4 ± 2.0 (67.2–76.7)	71.6 ± 1.7 (67.5–77.8)
Mineral (% of FFM)	5.7 ± 0.5 (4.2–7.1)	6.0 ± 0.6 (3.7–7.2)
Protein (% of FFM)	22.9 ± 1.8 (18.1–27.4)	22.3 ± 1.4 (17.5–26.3)
D_FFM_ (g·cm^−3^)	1.101 ± 0.008 (1.082–1.119)	1.102 ± 0.007 (1.077–1.118)

BMC = bone mineral content; BMD = bone mineral density; FFM = fat-free mass.

There were some differences by position for some of the team sports, quantified by Cohen’s *d*. Front line players were taller than setters and liberos for both men’s (187.6 ± 3.4 cm vs. 180.5 ± 5.4 cm, *d* = 1.69) and women’s (174.6 ± 5.2 cm vs. 165.3 ± 1.8 cm, *d* = 2.20) volleyball. Forwards (e.g. props and locks) were heavier and had higher %BF than backs (e.g. wings and centers) for both men’s (BM: 97.9 ± 13.7 kg vs. 83.1 ± 5.1 kg, *d* = 1.31; %BF: 21.7 ± 7.5% vs. 13.5 ± 2.6%, *d* = 1.32) and women’s (BM: 80.4 ± 11.9 kg vs. 62.4 ± 4.6 kg, *d* = 1.74; %BF: 31.9 ± 6.2% vs. 18.1 ± 2.0%, *d* = 2.57) rugby. All Cohen’s d values above were considered large-sized effects (*d* ≥ 0.80) [33].

## 4. Discussion

There are several key observations from this data collection. First, athletes are heterogeneous across sports with regard to height, BM, %BF, and FFMI; however, within a given sport, or more specifically position, there tends to be greater homogeneity of these variables. Athletes in weight-sensitive sports (e.g. climbing and cycling) in which moving BM against gravity is critical to success were the lightest and among the leanest. Athletes in which power is critical to success (e.g. powerlifting and rugby) were among the heaviest and had the highest FFMI. This finding is consistent with Silva’s [4] review of body composition phenotypes for athletic performance.

Several sports had both men’s and women’s teams. When the rules, events, or styles of play are similar between the men’s and women’s sports, the same body composition phenotypes are seen. For example, both men’s and women’s climbing, cycling, gymnastics, ultimate and volleyball athletes had team %BF values below the mean for their sex, while rugby and swimming had team %BF values above the mean for their sex. However, rules or events in some sports differ between the men’s and women’s teams, and these differences are reflected in the body composition phenotypes. For example, men’s lacrosse is a contact sport with checking allowed, but women’s lacrosse involves more finesse. This is reflected in the differences in body composition phenotypes between men’s and women’s lacrosse players. Men’s lacrosse players were among the heavier male athletes with above average FFMI. In contrast, women’s lacrosse players were below average in both BM and FFMI compared to other women’s clubs. Different patterns were also observed for rodeo athletes with females participating in roping and barrel racing and men competing in saddle bronc riding and steer wrestling. This may reflect the difference in team ranking for %BF, with women rodeo athletes slightly below the mean %BF of the women club sports but men rodeo athletes having the highest %BF of the men club sports. This finding is consistent with the only known physiological study on collegiate rodeo athletes [35] that noted steer wrestlers had higher %BF than several other athletic groups.

Historically, %BF has been the body composition variable of interest for coaches and researchers of athletes. However, FFM might be more relevant than %BF to successful athletic performance, particularly in strength and power sports [4]. Additionally, focusing on FFMI rather than %BF offers a more holistic approach to body composition assessment, shifting the emphasis toward increasing lean mass rather than decreasing FM. This might lead to healthier perceptions of body image and weight management strategies for some athletes; more research is needed on this topic. Recently, Jagim et al. [36] published a review of FFMI values for various NCAA sports, highlighting the increasing interest in FFMI

It is difficult to make comparisons to NCAA norms because many of the club sports do not exist within the NCAA, and the methodology to create the norms was different between studies (i.e. 4C model in the present study and DXA or ADP for NCAA norms). With these differences in mind, comparisons to norms created within the past 5 y of FFMI for NCAA athletes were made. The median FFMI for men in our study of club sports athletes was 21.0 kg·m^−2^, about 1 kg·m^−2^ less than the overall average of male NCAA athletes in the recent review by Jagim et al. [36]. The FFMI of our club sports men was less than the 22.8 kg·m^−2^ reported for a diverse sample of male NCAA athletes from 10 sports [5] or the 23.4 kg·m^−2^ for Division II or 24.3 kg·m^−2^ for Division I American football players [7], respectively. Brandner et al. [8] reported a mean FFMI of 23.4 kg·m^−2^ via ADP for a sample of NCAA Division III athletes from football, distance running, baseball, and wrestling. Close inspection of the descriptive data from these studies revealed NCAA athletes were considerably taller and heavier than our club sports sample. This is not surprising, as NCAA competition is a higher level than club sport. Further, most NCAA teams engage in either daily practice or conditioning nearly year round, whereas club sports teams practice only a few days per week and resistance training is not regimented. In contrast to the men, the median FFMI of 17.6 kg·m^−2^ for the women club sports athletes in the present study was nearly identical to the 17.5 kg·m^−2^ reported by Brandner et al. [8] and between the 17.0 kg·m^−2^ reported by Blue et al. [13] and 18.8 kg·m^−2^ reported by Harty et al. [6] in their studies of female NCAA athletes. Similarly, the overall average for women NCAA athletes in the Jagim et al. [36] review was 18.0 kg·m^−2^. Additionally, the mean heights, weights, and %BF were similar across these samples.

As stated in the introduction, an advantage of a 4C model assessment of body composition is improved accuracy, as components of the FFM are measured rather than assumed. Indeed, we observed significant differences in the D_FFM_ and fractional components of the FFM in this sample of athletes compared to Brozek’s [29] reference body assumptions. The percentage of water in the FFM was about 71.5%. Although this is much less than the 73.8% reference body [29], it is similar to the 70% measured by deuterium oxide in a recent sample of 80 collegiate and intramural athletes from a wide variety of sports [37]. Furthermore, their FFM hydration range of 63% to 78% is identical to what we observed. Similarly, the mineral fraction of the FFM in our sample (see Table 5) is much less than the assumed value of 6.8% [29], but it is similar to the 6.0% reported by Evans et al. [28] in their diverse sample of 84 NCAA Division I athletes. Thus, although the values for our FFM components differ from those of the reference body, the FFM composition of our club sports athletes is similar in some aspects to other 4C model studies of athletes from a variety of sports.

The focus of this investigation was on %BF and FFMI, not BMD; thus, lumbar and femoral neck DXA scans were not done. Nevertheless, some interesting observations were found from the total body BMD values. Cyclists had the lowest BMD of the 16 men’s clubs, and the sole female cyclist was among the low values for the women. Recent research showed low BMD to be highly prevalent among professional cyclists [38]. More research is needed to determine if low BMD is a problem among club cyclists as well as professionals. At the other end of the continuum, the highest team BMD values were observed in sports with frequent foot impacts (e.g. soccer and volleyball). The highest BMD for women was from a jump rope athlete with 14 y of competitive experience in the sport.

The strengths of this investigation include understudied club sports athletes and a large sample size for a 4C model study. To our knowledge, this is the first study to provide body composition data of university club sports athletes as opposed to NCAA athletes. Further, body composition data for some of these sports (e.g. racquetball, rodeo, pickleball, and ultimate) have rarely been reported in the literature. Numerous researchers have used the 4C model to measure the body composition of athletes; however, with the exception of Evans et al.’s [25] sample of 132 NCAA athletes, most 4C investigations have sample sizes of <30. To our knowledge, the present investigation is the largest 4C body composition study ever conducted on athletes.

There were several study limitations. First, although the total sample size was large, the sample sizes of some of the teams were too small to include in team comparisons. Additionally, the small sample size precluded making some positional comparisons within teams. Due to the small sample size, the percentile ranks and study results should be interpreted with caution. Second, despite providing athletes with pretesting instructions to achieve euhydration, approximately 10% of the sample had a USG > 1.025, suggestive of low hydration. Ideally, body composition assessment of athletes should occur when they are euhydrated [39]. However, this was not a major concern in the present study because TBW was included in the 4C measurement. Further, recent research shows that athletes with a high USG do not differ in TBW, the ratio of intra- to extra-cellular water, or the water fraction of the FFM when compared to those with a low USG [40]. Third, the traditional 4C body composition model uses hydrodensitometry and isotope dilution methods to measure BV and TBW, respectively [27]. For ease of measurement, we used ADP to measure BV and BIS to measure TBW. ADP is widely regarded as an acceptable method for measuring BV [41], and BIS was deemed a valid surrogate to isotope dilution for estimating TBW [42]. Additionally, previous research teams have used this contemporary approach of substituting ADP and BIS for hydrodensitometry and isotope dilution, respectively, in 3C and 4C studies of athletes [43–45]. However, ADP may underestimate the BV and %BF of lean athletes [46,47], and recent research is critical of substituting BIS for isotope dilution as the TBW measurement in multicomponent models [37].

In summary, this is the first known body composition study on collegiate club sports athletes. In general, there is considerable heterogeneity in body composition across sports, but homogeneity exists within a sport and specifically by position. Coaches and athletes can use this data to see what typical %BF and FFMI values are for club sports athletes and establish training goals to achieve levels of leanness and muscularity that are most advantageous for success in a given sport.

## Supplementary Material

Supplemental Material

## Data Availability

The dataset is publicly available on the figshare repository: https://doi.org/10.6084/m9.figshare.24094278.v1

## References

[1] American College of Sports Medicine. ACSM’s guidelines for exercise testing and prescription. 11^th^ ed. Philadelphia: Wolters Kluwer; 2022.

[2] Martinho DV, Naughton RJ, Faria A, et al. Predicting resting energy expenditure among athletes: a systematic review. Biol Sport. 2023;40(3):787–16. doi: 10.5114/biolsport.2023.119986 37398968 PMC10286600

[3] Ackland TR, Lohman TG, Sundgot-Borgen J, et al. Current status of body composition assessment in sport: review and position statement on behalf of the ad hoc research working group on body composition health and performance, under the auspices of the I.O.C medical commission. Sports Med. 2012;42(3):227–249. doi: 10.2165/11597140-000000000-00000 22303996

[4] Silva AM. Structural and functional body components in athletic health and performance phenotypes. Eur J Clin Nutr. 2019;73(2):215–224. doi: 10.1038/s41430-018-0321-9 30287933

[5] Currier BS, Harty PS, Zabriskie HA, et al. Fat-free mass index in a diverse sample of male collegiate athletes. J Strength Cond Res. 2019;33(6):1474–1479. doi: 10.1519/JSC.0000000000003158 30985525

[6] Harty PS, Zabriskie HA, Stecker RA, et al. Upper and lower thresholds of fat-free mass index in a large cohort of female collegiate athletes. J Sports Sci. 2019;37(20):2381–2388. doi: 10.1080/02640414.2019.1634964 31238804

[7] Trexler ET, Smith-Ryan AE, Blue MNM, et al. Fat-free mass index in NCAA division I and II collegiate American football players. J Strength Cond Res. 2017;31(10):2719–2727. doi: 10.1519/JSC.0000000000001737 27930454 PMC5438288

[8] Brandner CF, Harty PS, Luedke JA, et al. Sport differences in fat-free mass index among a diverse sample of NCAA division III collegiate athletes. J Strength Cond Res. 2022;36(8):2212–2217. doi: 10.1519/JSC.0000000000004267 35612943

[9] He X, Li Z, Tang X, et al. Age- and sex-related differences in body composition in healthy subjects aged 18 to 82 years. Med. 2018;97(25):e11152. doi: 10.1097/MD.0000000000011152 PMC6023800 29924020

[10] Ofenheimer A, Breyer-Kohansal R, Hartl S, et al. Reference values of body composition parameters and visceral adipose tissue (VAT) by DXA in adults aged 18–81 years—results from the LEAD cohort. Eur J Clin Nutr. 2020;74(8):1181–1191. doi: 10.1038/s41430-020-0596-5 32123345 PMC7402993

[11] Kirk B, Hassan EB, Brennan-Olsen S, et al. Body composition reference ranges in community-dwelling adults using dual-energy x-ray absorptiometry: the Australian body composition (ABC) study. J Cachexia Sarcopenia Muscle. 2021;12(4):880–890. doi: 10.1002/jcsm.12712 33991068 PMC8350202

[12] Kelly TL, Wilson KE, Heymsfield SB, et al. Dual energy x-ray absorptiometry body composition reference values from NHANES. PLOS ONE. 2009;4(9):e7038. doi: 10.1371/journal.pone.0007038 19753111 PMC2737140

[13] Blue MNM, Hirsch KR, Pihoker AA, et al. Normative fat-free mass index values for a diverse sample of collegiate female athletes. J Sports Sci. 2019;37(15):1741–1745. doi: 10.1080/02640414.2019.1591575 30893018

[14] Dobrosielski DA, Leppert KM, Knuth ND, et al. Body composition values of NCAA division 1 female athletes derived from dual-energy x-ray absorptiometry. J Strength Cond Res. 2021;35(10):2886–2893. doi: 10.1519/JSC.0000000000003213 31343559

[15] Fields JB, Metoyer CJ, Casey JC, et al. Comparison of body composition variables across a large sample of national collegiate athletic association women athletes from 6 competitive sports. J Strength Cond Res. 2018;32(9):2452–2457. doi: 10.1519/JSC.0000000000002234 29189580

[16] McKay AKA, Stellingwerff T, Smith ES, et al. Defining training and performance caliber: a participant classification framework. Int J Sports Physiol Perform. 2022;17(2):317–331. doi: 10.1123/ijspp.2021-0451 34965513

[17] Penninton B. Rise of college club teams creates a whole new level of success. The New York Times; 2008 Dec 2 [cited 2023 Dec 4]. Available from: https://www.nytimes.com/2008/12/02/sports/02club.html

[18] National Collegiate Athletic Association. NCAA sports sponsorship and participation rates report. 2023. Available from: https://ncaaorg.s3.amazonaws.com/research/sportpart/2022RES_SportsSponsorshipParticipationRatesReport.pdf

[19] Dopsaj M, Siljeg K, Milic R. Reference values and sensitivity for different body fat variables measured by bioimpedance method in female athletes in individual sports: discriminative and comparative study. Int J Morphol. 2023;41(3):717–724. doi: 10.4067/S0717-95022023000300717

[20] Bonilla DA, De Leon LG, Alexander-Cortez P, et al. Simple anthropometry-based calculations to monitor body composition in athletes: scoping review and reference values. Nutr Health. 2022;28(1):95–109. doi: 10.1177/02601060211002941 33792415

[21] Santos DA, Dawson JA, Matias CN, et al. Reference values for body composition and anthropometric measurements in athletes. PLOS ONE. 2014;9(5):e97846. doi: 10.1371/journal.pone.0097846 24830292 PMC4022746

[22] Wang ZM, Deurenberg P, Guo SS, et al. Six-compartment body composition model: inter-method comparisons of total body fat measurement. Int J Obes. 1998;22(4):329–337. doi: 10.1038/sj.ijo.0800590 9578238

[23] Lohman TG, Milliken LA, Sardinha LB. Introduction to body composition and assessment. In: Lohman T Milliken L, editors. ACSM’s body composition assessment. Champaign: Human Kinetics; 2020. p. 1–15.

[24] Heyward VH, Wagner DR. Applied body composition assessment. 2^nd^ ed. Champaign: Human Kinetics; 2004.

[25] Evans EM, Rowe DA, Misic MM, et al. Skinfold prediction equation for athletes developed using a four-component model. Med Sci Sports Exerc. 2005;37(11):2006–2011. doi: 10.1249/01.mss.0000176682.54071.5c 16286873

[26] Ducharme JB, Gibson AL, Mermier CM. Effect of predicted versus measured thoracic gas volume on body fat percentage in young adults. Int J Sport Nutr Exerc Metab. 2021;31(4):345–349. doi: 10.1123/ijsnem.2020-0342 34010808

[27] Wang ZM, Pi-Sunyer FX, Kotler DP, et al. Multicomponent methods: evaluation of new and traditional soft tissue mineral models by in vivo neutron activation analysis. Am J Cli Nut. 2002;76(5):968–974. doi: 10.1093/ajcn/76.5.968 12399267

[28] Evans EM, Prior BM, Arngrimsson SA, et al. Relation of bone mineral density and content to mineral content and density of the fat-free mass. J Appl Physiol. 2001;91(5):2166–2172. doi: 10.1152/jappl.2001.91.5.2166 11641358

[29] Brozek J, Grande F, Anderson JT, et al. Densitometric analysis of body composition: revision of some quantitative assumptions. Ann N Y Acad Sci. 1963;110(1):113–140. doi: 10.1111/j.1749-6632.1963.tb17079.x 14062375

[30] Efron B. Bootstrap methods: another look at the jackknife. Ann Statist. 1979;7(1):1–26. doi: 10.1214/aos/1176344552

[31] Efron B, Tibshirani RJ. An introduction to the bootstrap. Boca Raton: Chapman & Hall; 1993.

[32] Mooney CZ, Duval RD. Bootstrapping: a nonparametric approach to statistical inference. Newbury Park: SAGE; 1993.

[33] Cohen J. Statistical power analysis for the behavioral sciences. 2^nd^ ed. Hillsdale: Erlbaum; 1988.

[34] Tabachnick BG, Fidell LS. Using multivariate statistics. 3^rd^ ed. (NY): Harper Collins; 1996.

[35] Meyers MC, Wilkinson JG, Elledge JR, et al. Exercise performance of collegiate rodeo athletes. Am J Sports Med. 1992;20(4):410–415. doi: 10.1177/036354659202000408 1415883

[36] Jagim AR, Harty PS, Jones MT, et al. Fat-free mass index in sport: normative profiles and applications for collegiate athletes. J Strength Cond Res. 2024;38(9):1687–1693. doi: 10.1519/JSC.0000000000004864 39074219

[37] Cataldi D, Bennett JP, Quon BK, et al. Agreement and precision of deuterium dilution for total body water and multicompartment body composition assessment in collegiate athletes. J Nutr. 2022;152(9):2048–2059. doi: 10.1093/jn/nxac116 35665820

[38] Hilkens L, Van Schijndel N, Weijer V, et al. Low bone mineral density and associated risk factors in elite cyclists at different stages of a professional cycling career. Med Sci Sports Exerc. 2023;55(5):957–965. doi: 10.1249/MSS.0000000000003113 36595659 PMC10090358

[39] Meyer NL, Sundgot-Borgen J, Lohman TG, et al. Body composition for health and performance: a survey of body composition assessment practice carried out by the ad hoc research working group on body composition, health and performance under the auspices of the IOC medical commission. Br J Sports Med. 2013;47(16):1044–1053. doi: 10.1136/bjsports-2013-092561 24065075

[40] Francisco R, Jesus F, Nunes CL, et al. Athletes with different habitual fluid intakes differ in hydration status but not in body water compartments. Scand J Med Sci Sports. 2023;33(7):1072–1078. doi: 10.1111/sms.14355 36951582

[41] Fields DA, Goran MI, McCrory MA. Body-composition assessment via air-displacement plethysmography in adults and children: a review. Am J Clin Nutr. 2002;75(3):453–467. doi: 10.1093/ajcn/75.3.453 11864850

[42] Moon JR, Tobkin SE, Roberts MD, et al. Total body water estimations in healthy men and women using bioimpedance spectroscopy: a deuterium oxide comparison. Nutr Metab. 2008;5(1):article 7. doi: 10.1186/1743-7075-5-7 PMC2323003 18353180

[43] Hyde PN, Kendall KL, Fairman CM, et al. Use of B-mode ultrasound as a body fat estimate in collegiate football players. J Strength Cond Res. 2016;30(12):3525–3530. doi: 10.1519/JSC.0000000000001447 27861264

[44] Nickerson BS, Snarr RL, Ryan GA. Validity of foot-to-foot bioelectrical impedance for estimating body composition in NCAA division I male athletes: a 3-compartment model comparison. J Strength Cond Res. 2019;33(12):3361–3366. doi: 10.1519/JSC.0000000000002999 30789577

[45] Silva AM, Fields DA, Quiterio AL, et al. Are skinfold-based models accurate and suitable for assessing changes in body composition in highly trained athletes? J Strength Cond Res. 2009;23(6):1688–1696. doi: 10.1519/JSC.0b013e3181b3f0e4 19675495

[46] Moon JR, Eckerson JM, Tobkin SE, et al. Estimating body fat in NCAA division I female athletes: a five-compartment model validation of laboratory methods. Eur J Appl Physiol. 2009;105(1):119–130. doi: 10.1007/s00421-008-0881-9 18936958

[47] Peeters MW, Goris M, Keustermans G, et al. Body composition in athletes: a comparison of densitometric methods and tracking of individual differences. Eur J Sport Sci. 2013;13(1):78–85. doi: 10.1080/17461391.2011.606836

