# Supplementary figures and images for: Multicomponent body composition of university club sport athletes
Source: J Int Soc Sports Nutr. 2024 Dec 26;22(1):2446575. doi: 10.1080/15502783.2024.2446575 (PMC11703473; doi:10.1080/15502783.2024.2446575)

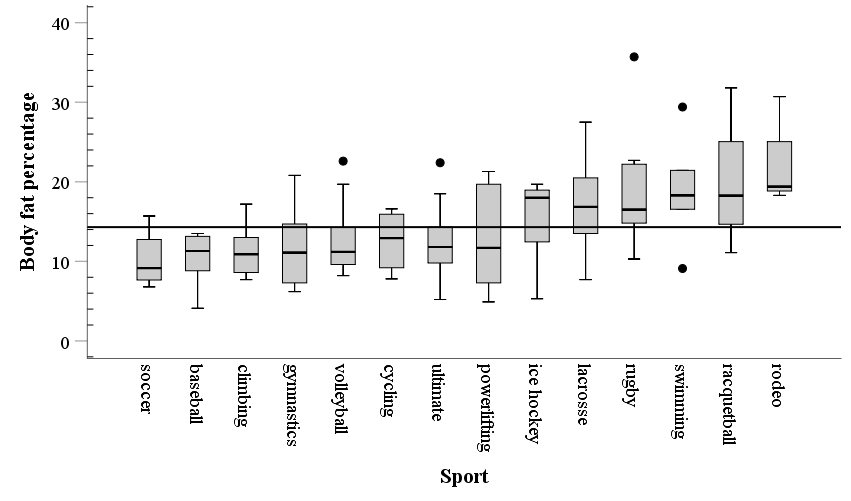

Supplement: Supplemental Material [file RSSN_A_2446575_SM0124.zip › Fig/Fig1_suppl.tif]

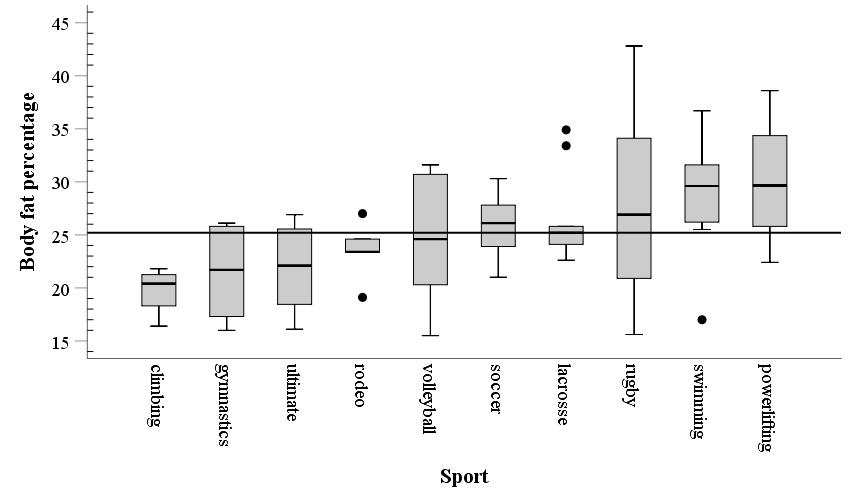

Supplement: Supplemental Material [file RSSN_A_2446575_SM0124.zip › Fig/Fig2_suppl.tif]

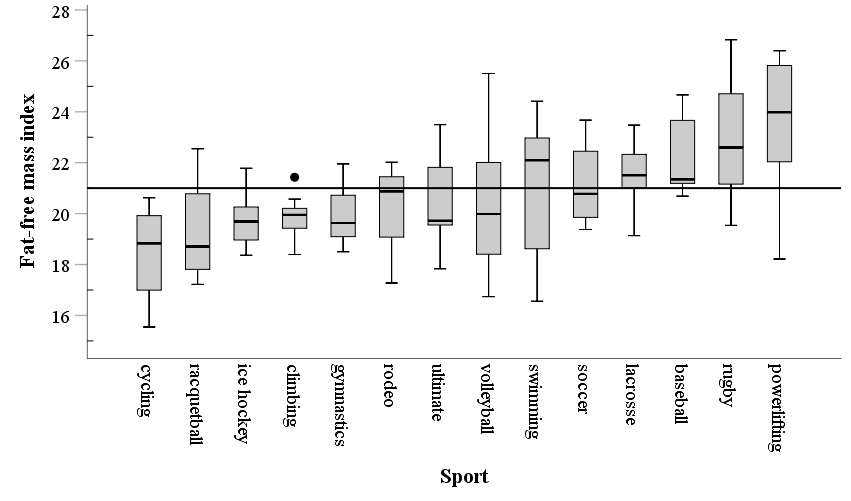

Supplement: Supplemental Material [file RSSN_A_2446575_SM0124.zip › Fig/Fig3_suppl.tif]

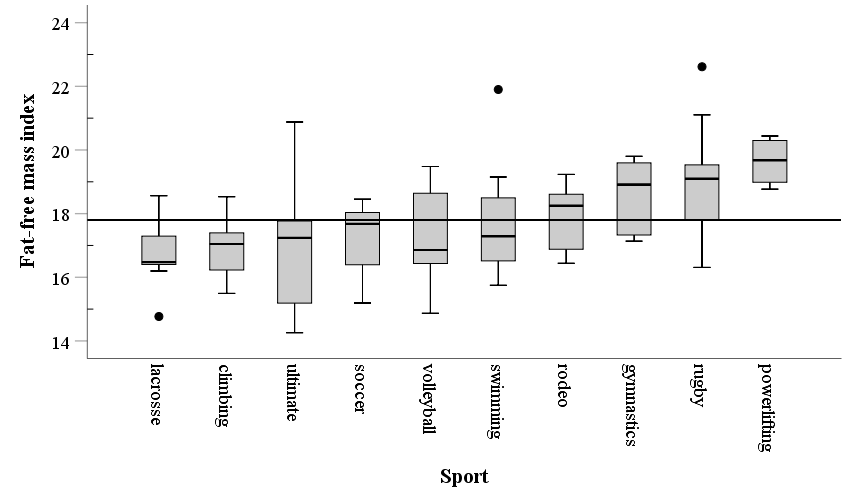

Supplement: Supplemental Material [file RSSN_A_2446575_SM0124.zip › Fig/Fig4_suppl.tif]
